# Association between migraine and suicidal behavior among Ethiopian adults

**DOI:** 10.1186/s12888-018-1629-7

**Published:** 2018-02-12

**Authors:** Hanna Y. Berhane, Bethannie Jamerson-Dowlen, Lauren E. Friedman, Yemane Berhane, Michelle A. Williams, Bizu Gelaye

**Affiliations:** 1 Addis Continental Institute of Public Health, Addis Ababa, Ethiopia; 2 Department of Epidemiology, Harvard T.H. Chan School of Public Health, Boston, MA USA

**Keywords:** Migraine, Suicidal behavior, Ethiopia

## Abstract

**Background:**

Despite the significant impact of migraine on patients and societies, few studies in low- and middle-income countries (LMICs) have investigated the association between migraine and suicidal behavior. The objective of our study is to examine the extent to which migraines are associated with suicidal behavior (including suicidal ideation, plans, and attempts) in a well-characterized study of urban dwelling Ethiopian adults.

**Methods:**

We enrolled 1060 outpatient adults attending St. Paul hospital in Addis Ababa, Ethiopia. Standardized questionnaires were used to collect data on socio-demographics, and lifestyle characteristics. Migraine classification was based on the International Classification of Headache Disorders-2 diagnostic criteria. The Composite International Diagnostic Interview (CIDI) was used to assess depression and suicidal behaviors (i.e. ideation, plans and attempts). Multivariable logistic regression models were used to estimate adjusted odds ratio (AOR) and 95% confidence intervals (95% CIs).

**Results:**

The prevalence of suicidal behavior was 15.1%, with a higher suicidal behavior among those who had migraines (61.9%). After adjusting for confounders including substance use and socio-demographic factors, migraine was associated with a 2.7-fold increased odds of suicidal behavior (AOR = 2.7; 95% CI 1.88–3.89). When stratified by their history of depression in the past year, migraine without depression was significantly associated with suicidal behavior (AOR: 2.27, 95% Cl: 1.49–3.46). The odds of suicidal behavior did not reach statistical significance in migraineurs with depression (AOR: 1.64, 95% CI: 0.40–6.69).

**Conclusion:**

Our study indicates that migraine is associated with increased odds of suicidal behavior in this population. Given the serious public health implications this has, attention should be given to the treatment and management of migraine at a community level.

## Background

Migraine is an under diagnosed and recurrent headache that is associated with sensitivity to light, nausea, or a reduced ability to function [1]. Migraine is a highly prevalent neurological disorder, affecting 1 out of every 10 individuals globally [1–3]. In Africa, though it is estimated that 5–10% of the population suffer from migraines [4, 5]; there is a scarcity of researches focusing on migraine.Higher prevalence have been reported among urban residents [4, 6], and women [5–8]. Earlier studies have also shown that migraine is higher among young adults (18–29) years [5].

Depending on the level of intensity during each episodes, migraine often has direct and immediate impact on patients’ daily activities. Its manifestations range from inability to lead a productive life due to loss of work/school days to withdrawal from social and leisure activities which ultimately affect the quality of life [9, 10]. Additionally, migraine disorders have been associated with psychiatric comorbidities including substance abuse, mood and anxiety disorders, depression, and suicidal behaviors [11–20]. Suicidal thoughts and behaviors (hereafter referred to as suicidal behaviors) which include suicidal ideation, plans, and attempts are predictors of completed suicide [21, 22].

Annually more than half a million people die due to suicide; while three-fourth of this deaths are in low and middle income countries (LAMICs) [23].Despite the profound personal, societal, and economic consequences both these problems have; knowledge pertaining to their association remain limited, particularly in LAMICs. Though previous research have shown the association between migraine and suicidal behaviors [16, 19, 20, 24]; no studies in sub-Saharan Africa have investigated this association. Due to the high burden of migraine in sub-Saharan Africa and the existing knowledge gap, we sought to evaluate the extent to which migraine headaches are associated with suicidal behaviors, including suicidal ideation, plan, and attempts, in a well-characterized study of urban dwelling Ethiopian adults.

## Methods

### Study design and population

This cross-sectional study enrolled 1060 participants attending the outpatient facility at the Saint Paul Hospital in Addis Ababa, Ethiopia. All patients evaluated in the internal medicine, general surgery and gynecological outpatient departments during the period of December to July 2011 were eligible to be included in the study. Those who were unable to communicate with the interviewers directly (those with diagnosed mental disabilities and hearing disabilities) were excluded. Interviewer administered structured questionnaires were used to collect data from all individuals who have consented to participate. Prior to data collection; the interviewers who were nurses by training were given an intensive training on: the contents of the questionnaire, interview techniques and ethical conduct of human subjects research. Continuous supervision and support was provided by the research coordinator throughout the period of data collection.

### Major depressive disorder and suicidal behavior

Major depressive disorder (MDD) and suicidal behavior were assessed using the depression module of the Composite International Diagnostic Interview (CIDI) 3.0 [25]. The CIDI is a fully-structured interview that assesses mental disorders according to the definitions and criteria of the ICD-10 [26] and DSM-IV [27]. For this study we used the DSM-IV definition of MDD: presence of five out of nine depressive symptoms that persist for 2 weeks or longer, are present for most of the day nearly every day, and cause significant distress or impairment [27].

Suicidal behavior was classified as ideation, attempt, and plan based on participant self-report [28]. Participants answered questions relevant to the presence of ideation, plan(s) and/or attempts during their most crucial depressive episode within the past year. In particular, the following questions were included: “During that period, did you ever think that it would be better if you were dead?”, “Did you make a suicide plan?” and “Did you make a suicide attempt?” if the respondent responded “Yes” to either of the three questions the individual was classified as having suicidal behaviors.

### Migraine disorders

For migraine, we used a structured migraine assessment questionnaire adapted from previously validated tool [29] . Migraine was classified according to the ICHD-II criteria [30]; it was defined by at least 5 lifetime headache attacks lasting 4–72 h, with at least 2 of the four qualifying pain characteristics (unilateral location, pulsating quality, moderate or severe pain intensity, aggravation by or causing avoidance of routine physical activity) and at least one of the associated symptoms (nausea and/or vomiting, or photo and photophobia).

### Other covariates

Structured questionnaires were used to collected data on socio-demographic characteristics, including sex, age (in years), and marital status (married, never married, other). Other socio-demographic covariates included: education (≤primary, secondary education, college graduate), smoking status (never, former, current), past year alcohol consumption (non-drinker, < once per month, ≥ 1 day per week), khat chewing (never, former, current), body mass index (BMI) (<18.5, 18.5–24.9, 24.9–29.9, ≥30 kg/m^2^), and self-reported physical and mental health status (excellent/very good/good vs. fair/poor).

### Statistical analysis

Data analyses was conducted using SPSS version 23.0 (IBM SPSS, Chicago, IL). Continuous variables were summarized as means (± standard deviation), and categorical variables as number and percentages. Multivariable logistic regression models were used to estimate odds ratios (ORs) and 95% confidence intervals (95% CI). Forward logistic regression modeling procedures combined with the change in-estimate approach were used to select the final multivariable adjusted models on the association between migraine and suicidal behaviors. Variables of a priori interest (e.g., age and sex) were included in final models. Previous studies have shown that depression is associated with suicidal behaviors and migraine. We therefore performed a sensitivity analysis stratifying our analysis by current(past year) depression status. Reported *p*-values are two-sided with a statistical significance set at *p* < 0.05.

## Results

A total of 1060 individuals with a mean age of 35.7 ± 12.1 years took part in this study. Of those, the majority were women (60%), married (51.3%) and had a low educational level (< grade 6) (44.7%). Current smoking and Khat use was reported by 4.1% and 20.6% of participants, respectively. When asked about their health status more than half of the participants reported having a good, very good, or excellent physical (56.2%) and mental health (65.9%). Percentage of those having depression (life time and in past year) was 24.7% while any suicidal behavior was 15.1% (Table 1).

**Table 1:** Socio-demographic and reproductive characteristics of the study population according to types of migraine (*N* = 1060)

Characteristics	All participants (N = 1060)		No migraine (*N* = 639)		Migraine (*N* = 421)		*P-*value
	*n*	%	*n*	%	*n*	%	
Age (years)^a^	35.68 ± 12.08		35.95 ± 12.10		35.28 ± 12.05		0.378
Sex							
Women	637	60.1	330	51.6	307	72.9	< 0.001
Men	423	39.9	309	48.4	114	27.1	
Marital Status							
Married	542	51.3	342	53.7	200	47.6	0.011
Never married	335	31.7	204	32.0	131	31.2	
Other	180	17.0	91	14.3	89	21.2	
Education							
≤ Primary (1–6)	474	44.7	248	38.8	226	53.7	< 0.001
Secondary (7–12)	357	33.7	233	36.5	124	29.5	
College graduate	229	21.6	158	24.7	71	16.9	
Smoking status							
Never	913	86.1	534	83.6	379	90.0	0.010
Former	104	9.8	76	11.9	28	6.7	
Current	43	4.1	29	4.5	14	3.3	
Alcohol consumption past year							
Non-drinker	601	56.7	340	53.2	261	62.0	0.002
< once a month	357	33.7	223	34.9	134	31.8	
≥ 1 day a week	102	9.6	76	11.9	26	6.2	
Khat chewing							
Never	783	73.9	455	71.2	328	77.9	0.052
Former	59	5.6	39	6.1	20	4.8	
Current	218	20.6	145	22.7	73	17.3	
Body mass index (kg/m^2^)							
< 18.5	174	16.5	111	17.4	63	15.1	0.252
18.5–24.9	629	59.7	373	58.6	256	61.4	
24.9–29.9	184	17.5	118	18.5	66	15.8	
≥ 30	67	6.4	35	5.5	32	7.7	
Self-reported physical health							
Excellent/very good/good	596	56.2	410	64.2	186	44.2	< 0.001
Poor/fair	464	43.8	229	35.8	235	55.8	
Self-reported mental health							
Excellent/very good/good	699	65.9	478	74.8	221	52.5	< 0.001
Poor/fair	361	34.1	161	25.2	200	47.5	
Depression (past year)	70	6.6	23	3.6	47	11.2	< 0.001
Depression (lifetime)	192	18.1	73	11.4	119	28.3	< 0.001
Suicidal behavior (any type) ^b^	160	15.1	61	9.5	99	23.5	< 0.001
Suicidal ideation	154	14.5	60	9.4	94	22.3	< 0.001
Suicidal plan	65	6.1	25	3.9	40	9.5	< 0.001
Suicidal attempt	44	4.2	16	2.5	28	6.7	0.001

Due to missing data, percentages may not add up to 100%

^a^Mean ± standard deviation (SD)

^b^Non-mutually exclusive subcomponents

For continuous variables, *P*-value was calculated using the one-way ANOVA; for categorical variables, *P*-value was calculated using the Chi-square test

Any suicidal behavior was reported by 160 (15.1%) study participants; which included suicidal ideation (14.5%), suicide plan (6.1%) or suicide attempt (4.2%). Characteristics of study participants by migraine status is also presented in Table 1. Migrainuers were more likely to be women, have less education and were more likely to report their physical or mental health status as fair or poor. Participants with migraine were also more likely to report depression (both past year and lifetime) and suicidal behaviors, including suicide ideation, plan, or attempt (*p* ≤ 0.001).

Characteristics of the study population according to suicidal behavior are shown in Table 2. Participants with any suicidal behavior were more likely to be women, married, and more likely to have self-reported fair/poor physical and mental health status. Participants with any suicidal behaviors were also more likely to have past year or lifetime depression (*p* < 0.01).

**Table 2:** Socio-demographic and reproductive characteristics of the study population according to suicidal behavior (*N* = 1060)

Characteristics	No suicidal behavior (*N* = 900)		Any suicidal behavior (*N* = 160)		*P-*value
	*n*	%	*n*	%	
Age (years)^a^	35.73 ± 12.13		35.42 ± 11.83		0.766
Sex					
Women	526	58.4	111	69.4	0.011
Men	374	41.6	49	30.6	
Marital Status					
Married	474	52.8	68	42.5	0.004
Never married	284	31.7	51	31.9	
Other	139	15.5	41	25.6	
Education					
≤ Primary (1–6)	402	44.7	72	45.0	0.850
Secondary (7–12)	301	33.4	56	35.0	
College graduate	197	21.9	32	20.0	
Smoking status					
Never	778	86.4	135	84.4	0.628
Former	85	9.4	19	11.9	
Current	37	4.1	6	3.8	
Alcohol consumption past year					
Non-drinker	499	55.4	102	63.7	0.098
< once a month	309	34.3	48	30.0	
≥ 1 day a week	92	10.2	10	6.3	
Khat chewing					
None	667	74.1	116	72.5	0.569
Former	52	5.8	7	4.4	
Current	181	20.1	37	23.1	
Body mass index (kg/m^2^)					
< 18.5	147	16.4	27	17.1	0.549
18.5–24.9	539	60.2	90	57.0	
24.9–29.9	157	17.5	27	17.1	
≥ 30	53	5.9	14	8.9	
Self-reported physical health					
Excellent/very good/good	537	59.7	59	36.9	< 0.001
Poor/fair	363	40.3	101	63.1	
Self-reported mental health					
Excellent/very good/good	631	70.1	68	42.5	< 0.001
Poor/fair	269	29.9	92	57.5	
Depression (past year)	21	2.3%	49	30.6	< 0.001
Depression (lifetime)	69	7.7	123	76.9	< 0.001

Due to missing data, percentages may not add up to 100%

^a^Mean ± standard deviation (SD)

For continuous variables, *P*-value was calculated using the one-way ANOVA; for categorical variables, *P*-value was calculated using the Chi-square test

The presence of migraine was associated with a 2.91-fold increased odds of suicidal behavior (OR: 2.91, 95% Cl: 2.06–4.12) as compared to participants without migraine. After adjusting for confounders including age, sex, education, and BMI, migrainuers were 2.71-times more likely to report suicidal behaviors compared to non-migrainuers (AOR:2.71, 95% CI: 1.89–3.89). The results remained similar after further adjusting for khat chewing and past year alcohol consumption (AOR: 2.70, 95% CI: 1.88–3.89). When life time history of depression was added to the model, the association between migraine and suicidal behaviors was greatly attenuated and became statistically insignificant (AOR: 1.49, 95% CI: 0.93–2.39) (Table 3).

**Table 3:** Association between migraine and suicidal behavior (N = 1060)

	No suicidal behavior (N = 900)		Any suicidal behavior (N = 160)					
	*n*	%	*n*	%	Unadjusted OR (95% CI)	Adjusted OR (95% CI)^a^	Adjusted OR (95% CI)^b^	Adjusted OR (95% CI) ^c^
No Migraine	578	64.2	61	38.1	Reference	Reference	Reference	Reference
Migraine	322	35.8	99	61.9	2.91 (2.06–4.12)	2.71 (1.89–3.89)	2.70 (1.88–3.89)	1.49 (0.93–2.39)

*Abbreviations*: *OR* odds ratio, *CI* confidence interval

^a^Adjusted for age (continuous), sex, education, and BMI categories

^b^Adjusted for age (continuous), sex, education, BMI categories, khat chewing, and past year alcohol consumption

^c^Adjusted for age (continuous), sex, education, BMI categories, khat chewing, past year alcohol consumption, and lifetime depression

We next explored the association between migraine and suicidal behavior after stratifying by past year depression status (Table 4). Among individuals without a history of depression (in the past year), the odds of suicidal behavior was 2-times higher among those with migraine as compared to those without migraine in a fully adjusted model (AOR: 2.27, 95% Cl: 1.49–3.46). Among individuals with a history of depression (in the past year), the odds of suicidal behavior was modestly increased but did not reach statistical significance for migraineurs as compared with those without migraine (AOR: 1.64 95% CI: 0.40–6.69).

**Table 4:** Association between migraine and suicidal behavior stratified by past year depression status (*N* = 1060)

Migraine Without Depression	No suicidal behavior (*N* = 879)		Any suicidal behavior (*N* = 111)				
	*n*	%	*n*	%	Unadjusted OR (95% CI)	Adjusted OR (95% CI)^a^	Adjusted OR (95% CI)^b^
No migraine	570	64.8	46	41.4	Reference	Reference	Reference
Migraine	309	35.2	65	58.6	2.61 (1.74–3.90)	2.27 (1.49–3.46)	2.27 (1.49–3.46)
Migraine	No suicidal behavior		Any suicidal behavior				
With Depression	(*N* = 21)		(*N* = 49)				
No migraine	8	38.1	15	30.6	Reference	Reference	Reference
Migraine	13	61.9	34	69.4	1.40 (0.48–4.07)	1.22 (0.36–4.13)	1.64 (0.40–6.69)

*Abbreviations*: *OR* odds ratio, *CI* confidence interval

^a^Adjusted for age (continuous), sex, education, and BMI categories

^b^Adjusted for age (continuous), sex, education, BMI categories, khat chewing, and past year alcohol consumption

## Discussion

Our results show that migraine is significantly associated with suicidal behaviors (including suicidal ideation, plan, or attempts), after adjusting for confounders including age, sex, BMI, education, khat use, and past year alcohol consumption. However, when lifetime depression was fitted in the model, a statistically significant association was not observed. Following further analysis to understand the effect of depression; it was found that migrainuers without history of depression in the past year had a 2.27-fold increased odds of suicidal behaviors as compared to non-migrainuers.

Migraine had a strong positive association with increased odds of suicidal ideation and attempts; this association has been also established from previous studies in high income countries including the US, Canada, Taiwan, Norway, Italy, and Korea [15, 20]. For example, a recent study among members of a Health Maintenance Organization in Michigan found that migraineurs had an increased risk of suicidal attempts during a 2 year follow-up [19]. The pooled analysis from a recent meta-analysis also found that migraine with aura was associated with increased odds of suicidal ideation (AOR: 1.31; 95% CI: 1.10–1.55), while no statistical association was observed for migraine without aura [31]. Similarly, suicidal attempts were found to be 3 to 7 times higher among those with migraines in a two- year follow up study [14, 16].

Chronic migraine is often comorbid with other conditions such as depression;a recent study in India found significant comorbidity between psychiatric disorders, including anxiety, depressive disorders, suicidality, and headache disorders [32]. Likewise, a study from Lima, Peru found that migrainuers without depression had an 1.8-fold increased odds of suicidal ideation while those with both migraine and depression had a 4.1 folds increased odds after adjusting for confounder [20]. This is contrary to our finding, adding history of life-time depression to the model resulted in no statically significant association between migraine and suicidal behaviors.However when we stratified individuals with their current (past year) depression status; participants with migraine and no depression has 2.27 fold increased odds of suicidal behavior (95% CI: 1.49–3.46). In line with this, Pompili et al. in their review found that the association of migraine and suicidal attempts was not necessarily due to coexting depression; but rather the chronic pain and loss of pleasure to engage in activities that is an independent risk factor for suicide [33].

Previous studies have documented the biological links between migraine and suicidal behaviors. Investigators suggest that the levels of cortisol and functioning of the hypothalamic-pituitary-adrenocortical (HPA) axis are affected by stressful events. Specifically, HPA activity has been found to be correlated with low grade cognitive stress in migraineurs [34]. Individuals with history of suicidal attempts were also found to have lower basal cortisol levels [35]. Furthermore in a study conducted among adolescent females, HPA-axis responses were associated to stress and risk of suicidal ideation [36]. Stressful events, including migraines, depression, and suicidal behaviors, may also be associated with serotonin levels. Changes in regulation and abnormalities of serotonergic mechanisms, including serotonin transporters, receptors, and metabolites have been associated with migraines and suicidal behaviors [37, 38]. Lastly chronic pain conditions, including migraine headaches, have been associated with suicidality [39–41]. Specifically, Ilgen et al. found an association between measures of head pain and suicidal ideation or attempts [40]. Chronic pain patients have an increased prevalence of suicidal ideation and attempts [42].

In the present study, there are a few limitations that should be considered. We used a cross-sectional study design which limits our inferences on the temporality between migraine and suicidal behaviors. Additionally, our hospital-based study population may limit our study findings generalizability to a broader general population. Our study used interviewer administered questionnaires in which the participants were asked questions about their physical and mental health, due to the social desirability bias participants may under-report their history of suicidal thought and behaviors and substance use. Migraine status could also be under reported as it could be affected by recall bias.

## Conclusion

Migraine is associated with increased odds of suicidal behaviors, including suicidal ideation, plans, and attempts, among urban Ethiopian adults. Studies should further investigate this comorbidity and possible risk factors for these disorders. Efforts should also be made to raise awareness about the burdens posed by migraine among the public as well as health professionals. In addition, health professionals should be aware of the comorbidity between migraine, depression, and suicidal thought and behaviors to implement effective screening and treatment of these comorbid disorders.

## Abbreviations

AOR: Adjust Odds Ratio
CI: Confidence Interval
CIDI: Composite International Diagnostic Interview
DSM-IV: Diagnostic and Statistical Manual of Mental Disorders, 4th Edition
ICD-10: International Classification of Diseases, 10th revision
ICHD-II: International Classification of Headache Disorders-2
LMICs: Low- and Middle-Income Countries
MMD: Major Depressive Disorder
OR: Odds Ratio

## References

[1] Charles A (2017). Migraine. N Engl J Med.

[2] Leonardi M, Raggi A (2013). Burden of migraine: international perspectives. Neurol Sci.

[3] Woldeamanuel YW, Cowan RP (2017). Migraine affects 1 in 10 people worldwide featuring recent rise: a systematic review and meta-analysis of community-based studies involving 6 million participants. J Neurol Sci.

[4] Woldeamanuel YW, Andreou AP, Cowan RP (2014). Prevalence of migraine headache and its weight on neurological burden in Africa: a 43-year systematic review and meta-analysis of community-based studies. J Neurol Sci.

[5] Gelaye B, Peterlin BL, Lemma S, Tesfaye M, Berhane Y, Williams MA (2013). Migraine and psychiatric comorbidities among sub-saharan african adults. Headache.

[6] Domingues R, Aquino C, Santos J, Silva A, Kuster G (2006). Prevalence and impact of headache and migraine among Pomeranians in Espirito Santo, Brazil. Arq Neuropsiquiatr.

[7] Smitherman TA, Burch R, Sheikh H, Loder E (2013). The prevalence, impact, and treatment of migraine and severe headaches in the United States: a review of statistics from national surveillance studies. Headache.

[8] Breslau N, Rasmussen BK (2001). The impact of migraine: epidemiology, risk factors, and co-morbidities. Neurology.

[9] Risal A, Manandhar K, Holen A, Steiner TJ, Linde M (2016). Comorbidities of psychiatric and headache disorders in Nepal: implications from a nationwide population-based study. J Headache Pain..

[10] Mannix S, Skalicky A, Buse DC, Desai P, Sapra S, Ortmeier B (2016). Measuring the impact of migraine for evaluating outcomes of preventive treatments for migraine headaches. Health Qual Life Outcomes.

[11] Buse D, Silberstein S, Manack A, Papapetropoulos S, RB L. (2013). Psychiatric comorbidities of episodic and chronic migraine. J Neurol.

[12] Bruti G, Magnotti MC, Iannetti G (2012). Migraine and depression: bidirectional co-morbidities?. Neurol Sci.

[13] Breslau N, Schultz L, Stewart W, Lipton R, Welch K (2001). Headache types and panic disorder: directionality and specificity. Neurology.

[14] Breslau NDG, Andreski P (1991). Migraine, psychiatric disorders, and suicide attempts: an epidemiologic study of young adults. Psychiatry Res.

[15] Novic A, Kolves K, O'Dwyer S, De Leo D (2016). Migraine and suicidal behaviors: a systematic literature review. Clin J Pain.

[16] Breslau N (1992). Migraine, suicidal ideation, and suicide attempts. Neurology.

[17] Breslau NDG, Schultz LR, Peterson EL (1994). Joint 1994 Wolff award presentation. Migraine and major depression: a longitudinal study. Headache.

[18] Breslau N, Lipton R, Stewart W, Schultz L, Welch K (2003). Comorbidity of migraine and depression: investigating potential etiology and prognosis. Neurology.

[19] Breslau N, Schultz L, Lipton R, Peterson E, Welch K (2012). Migraine headaches and suicide attempt. Headache.

[20] Friedman LE, Gelaye B, Rondon MB, Sanchez SE, Peterlin BL, Williams MA (2016). Association of Migraine Headaches with Suicidal Ideation among Pregnant Women in lima. Peru Headache.

[21] Nock MK, Borges G, Bromet EJ, Cha CB, Kessler RC, Lee S (2008). Suicide and suicidal behavior. Epidemiol Rev.

[22] Whittier AB, Gelaye B, Deyessa N, Bahretibeb Y, Kelkile TS, Berhane Y (2016). Major depressive disorder and suicidal behavior among urban dwelling Ethiopian adult outpatients at a general hospital. J Affect Disord.

[23] Saxena S, Krug EG, Chestnov O and World Health Organization, eds. Preventing Suicide: A Global Imperative. Geneva: World Health Organization; 2014.

[24] Wang S, Fuh J, Juang K, Lu S (2009). Migraine and suicidal ideation in adolescents aged 13 to 15 years. Neurology.

[25] (WHO) WHO (1990). Composite international diagnostic interview (CIDI).

[26] (WHO) WHO (2004). International statistical classification of diseases and health related problems: (the) ICD-10.

[27] American Psychiatric Association, American Psychiatric Association. Task Force on DSM-IV. Diagnostic and statistical manual of mental disorders: DSM-IV-TR. 4.th ed. Washington, DC: American Psychiatric Association; 2000.

[28] Nock MK, Hwang I, Sampson NA, Kessler RC (2010). Mental disorders, comorbidity and suicidal behavior: results from the National Comorbidity Survey Replication. Mol Psychiatry.

[29] Samaan Z, Macgregor EA, Andrew D, McGuffin P, Farmer A (2010). Diagnosing migraine in research and clinical settings: the validation of the structured migraine interview (SMI). BMC Neurol.

[30] Society HCCotIH (2013). The international classification of headache disorders, 3rd edition (beta version). Cephalalgia.

[31] Friedman LE, Gelaye B, Bain PA, Williams MA. A systematic review and meta-analysis of migraine and suicidal ideation. Clin J Pain. 2016;33(7):659-65. 10.1097/AJP.0000000000000440 PMC5357206 27648590

[32] Desai SD, Pandya RH (2014). Study of psychiatric comorbidity in patients with headache using a short structured clinical interview in a rural neurology clinic in western India. J Neurosci Rural Pract.

[33] Pompili M, Di Cosimo D, Innamorati M, Lester D, Tatarelli R, Martelletti P (2009). Psychiatric comorbidity in patients with chronic daily headache and migraine: a selective overview including personality traits and suicide risk. J Headache Pain.

[34] Leistad RB, Stovner LJ, White LR, Nilsen KB, Westgaard RH, Sand T (2007). Noradrenaline and cortisol changes in response to low-grade cognitive stress differ in migraine and tension-type headache. J Headache Pain.

[35] Keilp JG, Stanley BH, Beers SR, Melhem NM, Burke AK, Cooper TB (2016). Further evidence of low baseline cortisol levels in suicide attempters. J Affect Disord.

[36] Giletta M, Calhoun C, Hastings P, Rudolph K, Nock M, Prinstein M (2015). Multi-level risk factors for suicidal ideation among at-risk adolescent females: the role of hypothalamic-pituitary-adrenal Axis responses to stress. J Abnorm Child Psychol.

[37] Pandey G (2013). Biological basis of suicide and suicidal behavior. Bipolar Disord.

[38] Gupta S, Nahas SJ, Peterlin BL (2011). Chemical mediators of migraine: preclinical and clinical observations. Headache.

[39] Ilgen M, Kleinberg F, Ignacio R, Bohnert A, Valenstein M, McCarthy J (2013). Noncancer pain conditions and risk of suicide. JAMA Psychiatry.

[40] Ilgen MA, Zivin K, McCammon RJ, Valenstein M (2008). Pain and suicidal thoughts, plans and attempts in the United States. Gen Hosp Psychiatry.

[41] Ratcliffe G, Enns M, Belik S, Sareen J (2008). Chronic pain conditions and suicidal ideation and suicide attempts: an epidemiologic perspective. Clin J Pain.

[42] Tang NK, Crane C (2006). Suicidality in chronic pain: a review of the prevalence, risk factors and psychological links. Psychol Med.

